# Aqueous extract of *Parkia biglobosa* (Jacq.) R. Br. (Fabaceae) exerts antiepileptogenic, anti-amnesic, and anxiolytic-like effects in mice *via* mechanisms involving antioxidant and anti-inflammatory pathways

**DOI:** 10.3389/fphar.2022.995881

**Published:** 2022-10-24

**Authors:** Antoine Kavaye Kandeda, Soline Menvouta, Symphorien Talom Mabou, Jonas Kouamouo, Théophile Dimo

**Affiliations:** ^1^ Department of Animal Biology and Physiology, University of Yaoundé I, Yaoundé, Cameroon; ^2^ Department of Pharmacy, University of the Mountains, Bangangté, Cameroon

**Keywords:** *Parkia biglobosa*, epileptogenesis, GABA, oxidative stress, neuroinflammation

## Abstract

*Parkia biglobosa* (Jacq.) R. Br. (Fabaceae) is a widely distributed tree, used in traditional medicine to treat amebiasis, hookworm infection, ascariasis, asthma, sterility, dental pain, headaches, cardiac disorders, and epilepsy. To date, no study on the effect of an aqueous extract of *P. biglobosa* on epileptogenesis and associated neuropsychiatric disorders has been undertaken. Therefore, this study aimed to investigate antiepileptogenic-, antiamnesic-, and anxiolytic-like effects of an aqueous extract of *P. biglobosa* using pentylenetetrazole (PTZ)-induced kindling in mice. Animals were divided into six groups of eight mice each. Thus, a PTZ group received distilled water (10 ml/kg, *per o*s), a positive control group received sodium valproate (300 mg/kg, *p.o*.), and three test groups received the aqueous extract of *P. biglobosa* (80, 160, and 320 mg/kg, *p.o*.).In addition, a control group of eight mice receiving distilled water (10 ml/kg, *p.o*.) was formed. The treatments were administered to mice, 60 min before administration of PTZ (20 mg/kg, i.p.). These co-administrations were performed once daily, for 22 days. The number and duration of seizures (stages 1, 2, 3, and 4 of seizures) exhibited by each mouse were assessed for 30 min during the treatment period. Twenty-four hours following the last administration of the treatments and PTZ, novel object recognition and T-maze tests were performed to assess working memory impairment in mice, while the open field test was performed to assess anxiety-like behavior. After these tests, the animals were sacrificed, and the hippocampi were collected for biochemical and histological analysis. During the period of PTZ-kindling, the extract at all doses completely (*p* < 0.001) protected all mice against stages 3 and 4 of seizures when compared to sodium valproate, a standard antiepileptic drug. The extract also significantly (*p* < 0.001) attenuated working memory impairment and anxiety-like behavior. In post-mortem brain analyses, the extract significantly (*p* < 0.001) increased γ-aminobutyric acid (GABA) level and reduced oxidative stress and inflammation. Histological analysis showed that the aqueous extract attenuated neuronal degeneration/necrosis in the hippocampus. These results suggest that the extract is endowed with antiepileptogenic-, anti-amnesic-, and anxiolytic-like effects. These effects seem to be mediated in part by GABAergic, antioxidant, and anti-inflammatory mechanisms. These results suggest the merit of further studies to isolate the bioactive molecules responsible for these potentially therapeutically relevant effects of the extract.

## 1 Introduction

Temporal lobe epilepsy (TLE) is a common and drug-resistant form of epilepsy in adults (Kandeda et al., 2022a). It is characterized by recurrent seizures that originate from a group of neurons and can spread to different parts of the brain (Kandeda et al., 2021a; Kandeda et al., 2022ab). TLE can be idiopathic, or it can result from brain damage caused by other pathologies (Nayak and Bandyopadhyay, 2022). Symptomatic TLE typically develops in three phases: 1) initial brain damage; 2) a latency phase, termed epileptogenesis, or silent period; and 3) the appearance of spontaneous seizures which become drug-resistant over time (Dhir, 2012; Kandeda et al., 2021a). Epileptogenesis is a pathogenic process in which modifications occur in the brain after a precipitating injury (prolonged febrile seizure, traumatic brain injury, intracerebral hemorrhage, stroke, *status epilepticus*, or infection) that leads to spontaneous recurrent seizures (Dhir, 2012; Kandeda et al., 2021a). These seizures result from a cascade of post-injury changes in the structure and function of neurons that are then physiological and morphological substrates of seizure activity (Dhir, 2012; Kandeda et al., 2021a). Structural changes, infections, genetic mechanisms, immune factors, and metabolic activities, alone or in combination, can lead to epileptogenesis and TLE. At the molecular and cellular levels, numerous reports implicate a deficit in GABA neurotransmission, an increase in oxidative stress, and inflammation as factors associated with neuronal loss in the hippocampus during TLE development (Hinton and Johnston, 2018; Kandeda et al., 2022d). Behaviorally, these alterations are particularly associated with cognitive and psychiatric disorders such as anxiety disorders and working memory impairment (Scott et al., 2017; Vinti et al., 2021). TLE is most frequently modeled in rodents to explore the mechanisms of epileptogenesis. Of all chemicals used to study epileptogenesis, chronic administration of subconvulsant doses of pentylenetetrazole (PTZ), an antagonist of γ-aminobutyric acid (GABA) receptor A, is used to closely mimic all of the neuropathological states of epileptogenesis in human (Shimada and Yamagata, 2018; Singh et al., 2021). PTZ kindling is a chronic model of TLE where intermittent and repetitive administration of subconvulsant doses of PTZ (20–40 mg/kg) can lead to progressive seizures severity, culminating in generalized tonic-clonic seizures in rodents (Dhir, 2012; Kandeda et al., 2021a). In Africa, many reports outline the use by traditional healers of medicinal plants with anticonvulsant activities (Auditeau et al., 2019; Suleiman et al., 2022).

A majority of the African population uses only these plant-based remedies to treat epilepsy since they do not have access to the conventional antiepileptic drugs used in modern therapies (Suleiman et al., 2022). These conventional antiepileptic drugs are often associated with cognitive and behavioral toxicities (Chen et al., 2017; Tomson et al., 2019; Beghi and Beghi, 2020), and they are not universally effective. In addition, drug resistance often develops during the use of conventional drugs to treat TLE (Salman et al., 2017). Thus, there is a need to develop new therapeutic agents (Mehla et al., 2017), and medicinal plants provide a lucrative source.

*Parkia biglobosa* (Jacq.) R. Br. (Fabaceae) is a leguminous tree that grows widely in west Africa and the northern part of Cameroon, and it is commonly called “*néré*" in the Malinké language of the Ivory Coast (Grønhaug et al., 2008; Burlando et al., 2019). In addition to its use in traditional medicine to treat epilepsy, the stem bark extract of *P. biglobosa* is also used to treat amebiasis, hookworm infection, ascariasis, asthma, ear complaints sterility, bronchitis, peptic ulcers, dental pain, pneumonia, bilharzia, fever, malaria, leprosy, and cardiac disorders (Grønhaug et al., 2008; Burlando et al., 2019).

Several studies have documented antihypertensive, antidiabetic, anxiolytic, nootropic, and neuroprotective properties of extracts from the plant (Oyedemi et al., 2021; Saleh et al., 2021). All of these properties are likely due to the presence of multiple groups of secondary metabolites in this plant, such as tannins, saponins, alkaloids, flavonoids, anthraquinones, mucilage, and triterpenoids (Kouadio et al., 2000; Komolafe et al., 2014; Bari et al., 2016). These compounds are endowed with antioxidant and anti-inflammatory activities (Kouadio et al., 2000; Komolafe et al., 2014; Bari et al., 2016), and have been proven elsewhere to be effective against anxiety, sedation, psychosis, nociception, and epilepsy (Kouadio et al., 2000; Komolafe et al., 2014; Bari et al., 2016). High-performance liquid chromatography (HPLC) analysis of an aqueous extract of *P. biglobosa* stem bark revealed six peaks which correspond to the presence of alkaloids, flavonoids, tannins, saponins, phenols, and anthraquinones (Yahaya et al., 2014). In previous studies, an aqueous extract of *P. biglobosa* stem bark has been shown to possess an anxiolytic-like effect in the elevated zero maze, open field, and elevated plus maze tests (Udobi and Onaolapo, 2012; Yahaya et al., 2014). This extract was also shown to improve the spatial memory of rats in the Y-maze test (Udobi and Onaolapo, 2012; Yahaya et al., 2014). The median oral lethal dose (LD_50_) of an aqueous extract of the stem bark of *P. biglobosa* was greater than 5,000 mg/kg body weight in rats, indicating its relative lack of toxicity, which is consistent with its wide use in folk medicine to manage neuropsychiatric disorders (Builders et al., 2019). Literature-based evidence led us to hypothesize that the *P. biglobosa* extract could be effective against epileptogenesis and associated neuropsychiatric disorders such as amnesia and anxiety. The current study evaluates these effects in the PTZ-induced kindling model of TLE in mice. The involvement of GABA neurotransmission, antioxidant defense, and anti-inflammatory actions during epileptogenesis was also investigated.

## 2 Materials and methods

### 2.1 Plant material and extract preparation

The plant material used in this work is the stem bark of *P. biglobosa* (Fabaceae), collected in Mora (Far North region of Cameroon), on the 28th of January 2019, early in the morning (5 h 30 am–7 h 00 am). The plant was identified by the National Herbarium of Cameroon (HNC) in comparison with a sample kept under reference No 58972/HNC. The plant name was checked with http://www.theplantlist.org/tpl1.1/record/ild-25404. The traditional therapist’s preparation protocol was followed in this study. The harvested barks of *P. biglobosa* were washed with tap water and dried. They were then pulverized to obtain a fine powder. A decoction of the plant was prepared by placing 60 g of dry powder from the barks of P. biglobosa into a container (2.5 L of distilled water). The mixture was brought to a boil for 30 min on a hotplate at 100 °C. After cooling, the mixture was filtered through Whatman No. 1 paper, then the resulting filtrate was lyophilized (freeze-dried) to avoid fermentation and hydrolysis in the extract. A dried mass of 4.64 g was obtained, i.e. a yield of 7.74%. The therapeutic dose recommended by the traditional healer was 160 mg/kg. This dose was surrounded by 80 mg/kg (dividing the therapeutic dose by 2) and 320 mg/kg (multiplying the therapeutic dose by 2).

### 2.2 Animals and bioethics

The animals used in this study were adult male mice of the *Mus musculus* strain, 2–3 months old, weighing between 20 and 30 g. These animals were raised in the animal facilities of the Laboratory of Animal Physiology (University of Yaoundé I, Cameroon). They were kept in cages (30 cm × 30 cm) with three animals per cage, under a natural cycle of 12 h of light/12 h of darkness, in an environment of approximately 25°C. These mice were given free access to drinking water and food. Before the beginning of the experiments, the animals were acclimatized for a week in the laboratory. Animal procedures were carried out following the guidelines of the Institutional Ethics Committee of the Cameroon Ministry of Scientific Research and Technological Innovation (Reg. no. FWA-IRD 0001954, 04/09/2006), which adopted the guidelines of the European Union on Animal Care (C.E.E. Council 86/609). The euthanasia of animals was performed according to the American Veterinary Medical Association (AVMA) guidelines for the euthanasia of animals. All animal studies, including allocation of animals to experimental groups, outcomes, experimental dosages, and statistical methods detailed here were carried out following the ARRIVE guidelines (https://www.nc3rs.org.uk/arriveguidelines/resources/author-checklists). The size of the sample used in the present study was determined based on previous laboratory findings (Bertoglio et al., 2017; Kandeda et al., 2022a; Kandeda et al., 2022b). This size was equally determined by ‘’Power G″ software, and the following formulae was used (Lan et al., 2010): sample size = (Z—score)2 * StdDev*(1—StdDev)/(margin of error) *2. Z: confident level and StdDev: standard deviation.

### 2.3 Chemicals

Pentylenetetrazole, sodium valproate, ninhydrin, trichloroacetic acid, hydrochloric acid, copper, tartaric acid, bromocresol, xylene, Ellmann’s reagent, thiobarbituric acid, and tris reagent were purchased from Sigma Chemical Lab. (Sigma, St. Louis, MO, United States).

### 2.4 Experimental design and PTZ-kindling

Kindling is the repetition of chemical or electrical stimulation of the brain, which initially does not induce seizures, but rather lowers the threshold of seizures (Dhir, 2012). It causes progression from simple partial seizures to generalized tonic-clonic seizures (Dhir, 2012).

On the first day of the experiments, the mice were marked, weighed, and divided into six groups of eight animals each as follows: normal group (named control group) only treated with distilled water (10 ml/kg; *per os*); negative control group (named PTZ group) treated with distilled water (10 ml/kg; *p.o*.); three test groups (named PB80, PB160, and PB320 groups) treated with the extract of *P. biglobosa* (80, 160, and 320 mg/kg, respectively, *p.o.*); and positive control group (named SV group) treated with sodium valproate (300 mg/kg; *p.o.*). The dose of sodium valproate used in the current study was chosen based on previous laboratory findings and preliminary screening with a median effective dose (ED_50_) determined (Kandeda et al., 2021a; Kandeda et al., 2022a).

One hour following the administration of the above-mentioned treatments, PTZ (20 mg/kg; i.p.) was injected intraperitoneally to all mice, except those of the control group only treated with distilled water (10 ml/kg; i.p.). These co-administrations (PTZ and different treatments) were repeated once daily, until the development of stage 4 seizures, on day 22. During this period, each animal was individually observed for 30 min, for the occurrence of seizures. Twenty-four hours following the end of the PTZ-kindling procedure, i.e. 23rd day, animals were challenged with PTZ (20 mg/kg; i.p.) to confirm that stage 4 of seizures had been reached in PTZ group animals. The PTZ-kindling model is the best model to appreciate the evolution of the epileptogenesis process in rodents. Therefore, epileptogenesis is completed when animals exhibit either stage 4 or 5 of seizures (Dhir, 2012; Kandeda et al., 2021b). Thus, stage 4 of seizures in two successive trials, during the kindling process, is widely sufficient to obtain complete epileptogenesis (Dhir, 2012; Kandeda et al., 2021b). In the present study, when animals reached stage 5, they immediately died.

The number and duration of eventual seizures were recorded according to Racine scale (Racine, 1972): stage 1 (hyperactivity, twitching, and/or convulsions); stage 2 (head shaking, nodding, and myoclonic convulsions); stage 3 (unilateral convulsions of the forelimbs); stage 4 (recovery with bilateral convulsions of the forelimbs); and stage 5 (generalized tonic-clonic convulsions with loss of the righting reflex).

**FIGURE 1:**
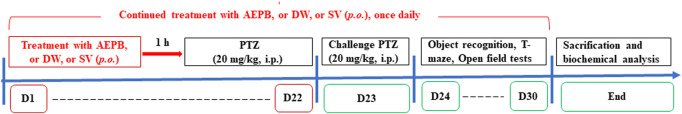
Experimental design of the study. AEPB, aqueous extract of *Parkia biglobosa*; DW, distilled water; SV, sodium valproate; D, day; PTZ, pentylenetetrazole.

Twenty-four hours following the last seizure procedure, i.e., on the 24th day, the object recognition test was performed for 3 days. Twenty-four hours later, i.e., on the 27th day, the T-maze task was also performed for 3 days. Finally, the open field test was performed on day 30 for 1 day (Figure 1). During the behavioral assessment which was conducted between 8:00 pm and 12:00 pm, the treatments were continued till the end of the assessment. Then the mice were sacrificed and the hippocampus was removed for biochemical and histological analyses. The experimenters were blind to the treatments, and behavioral assays were videotaped.

### 2.5 Behavioral assessment

#### 2.5.1 Object recognition task

This test performed on days 24, 25, and 26, aimed to assess the learning and memory of the form and color of an object. This test was performed as previously described by Kandeda et al. (2021c). This test took place in three phases, which correspond to the 3 days of the experiment: phase one corresponds to the habituation phase (day 24). Here, animals are individually allowed to explore the open field in the absence of objects; phase two corresponds to the familiarization phase (day 25), and each animal was allowed to explore, for 5 min, two identical objects (A + A) placed in symmetrical positions; phase three corresponds to the test phase (day 26). Thus, one of the old objects (A) is now replaced by a new object (B), and each animal was observed for 5 min exploration. The exploration time of each object was recorded, and the recognition or discrimination index was calculated as described by Kandeda et al. (2021c).

#### 2.5.2 T-maze task

This test was performed on days 27, 28, and 29. It consisted of a start compartment and two finish lanes, measuring each 30 cm long, 10 cm wide, and 25 cm high. Opaque guillotine doors were located at the exit of the start arm as well as at the entrance to each arrival arm (Djeuzong et al., 2021). A feeder of 7 cm in diameter and 1 cm high containing a food enhancer was placed at the end of each arrival arm. The experiment took place in three phases (corresponding to 3 days) as follows:

- The habituation phase (day 27). This consisted of familiarizing each mouse with the maze for a period of 5 min. A food enhancer was placed in each arrival arm to aid exploration. Thus, the animal was placed at the end of the starting arm. The experimenter opened all the guillotine doors and the animal chose one of the lanes of the arrival arms, indicating its preference. After the 5 min of observation, the mouse was returned to its cage, and the experimental device was cleaned with 70% ethanol to eliminate odors left by the next mouse;
- The acquisition phase (day 28). This consisted of repeating the same experience, but in this case, the discriminated arm was blocked by the guillotine;
- The retention phase (day 29) was similar to the habitation phase, but this time, the preferred arm is known beforehand. During this last phase, the number of entries and the time spent in the different arms (preferred and discriminated) of the maze were recorded and used to calculate percentages.

#### 2.5.3 Open field test

On day 30 of the experiment, the open field test was performed. This test is used to assess the level of locomotor activity, level of exploration, and emotional reactivity in rodents (Seibenhener and Wooten, 2015; Börchers et al., 2022). Therefore, the open field test represents a reliable measure of anxiety-like behavior in drug-treated and genetically manipulated rodents (Seibenhener and Wooten, 2015; Kraeuter et al., 2019). It was carried out following the method of Kandeda et al. (2022a). The open-field device has an area of 40 cm × 40 cm large, and a height of 45 cm. The exploration surface was made up of 16 squares of equal dimensions (5 cm × 5 cm) dividing the interior surface and 1 central square (5 cm × 5 cm). The test involved placing the animal in the center of the device so that it can explore freely. The number of recoveries, the time spent in the center of the arena, and the number of rearings (when the animal was standing on its hind legs on the edges of the experimental device) were recorded for a period of 5 min for each animal. At the end of each observation, the mouse was returned to its cage and the device was cleaned with 70% ethanol.

### 2.6 Sacrifice and preparation of homogenates

Immediately after the open field test, all animals were sacrificed by cervical dislocation.

The brains were removed, washed in 0.9% NaCl, blotted, weighed, and placed in beakers containing frozen 0.9% NaCl for solidification. After solidification, the organs were dissected-out to extract the hippocampus. The biochemical parameters were only evaluated in the hippocampus because this structure of the brain is mainly involved in seizure generation, i.e., the foci of seizures in the temporal lobe epilepsy (Das et al., 2009; Kandeda et al., 2022c)**.** Some hippocampi (*n* = 5) were homogenized into a ceramic mortar containing 50 mM Tris-Hcl buffer (pH 7.4) for the preparation of 10% homogenates. The obtained mixture was then centrifuged at 3,000 rpm for 15 min. The supernatant was recovered and used for neurochemical assays. Furthermore, the rest of the hippocampi (*n* = 3) were fixed in 10% formalin for further histological analyses.

### 2.7 Biochemical analyses

#### 2.7.1 Determination of γ-aminobutyric acid level

The amount of GABA contained in the hippocampus was evaluated according to the method of Lowe et al. (1958). In a basic medium, the reaction between ninhydrin and GABA gives a purplish red color which absorbs at 530 nm. The reagent was composed of a mixture of 0.2 mL of 0.14 M ninhydrin (solution prepared in bicarbonate buffer) and 0.1 mL of 10% trichloroacetic acid. Then, a 100 µL sample of homogenate was added to the reagent. This mixture was incubated at 60°C in a water bath for 30 min. After cooling, 5 ml of copper (II) tartrate solution (0.012 M) was added and the mixture was stirred at 25°C for 10 min. The resulting color intensity was measured using a spectrophotometer (Yoke Instrument Co. Ltd, Shangai, China). The determination of the level of GABA was made from a calibration curve, obtained by dissolving different masses of GABA (50, 100, 150, 200, 250, 300, 350, and 400 µg) mixed with 0.2 mg of glutamate in 1 mL of 10% trichloroacetic acid (Sutton and Simmonds, 1974).

#### 2.7.2 Determination of γ-aminobutyric acid-transaminase activity

The GABA-T activity was determined by the colorimetric method of Nayak and Chatterjee (2001). In the presence of iron chloride, semialdehyde succinic acid and 3-methyl-2-benzothiazolone-2-hydrazone form a colored complex that absorbs at 610 nm. Fifteen µmol of α-oxoglutarate, 15 µmol of GABA, 10 µg of pyridoxal phosphate, 0.1 mL of homogenate supernatant, and 0.1 mL of 5% methanol were placed into test tubes. The final volume of the mixture was made up with Tris buffer (50 mM HCl; 150 mM KCl; pH 7.4). The tubes were then incubated for 30 min at 37°C. The reaction was terminated by adding 0.5 mL of 20% trichloroacetic acid. Before reading, 0.3 mL of 12% FeCl_3_ was added to each tube. The absorbance of the complex formed by semialdehyde succinic acid and 3-methyl- 2-benzothiazolone-2-hydrazone (added chromogenic reagent) was read at 610 nm after 30 s and 90 s against the blank. The activity of GABA-T was calculated in pg/min/mg of tissue.

#### 2.7.3 Determination of L-glutamate decarboxylase activity

The activity of L-GAD was determined by using the colorimetric method of (Nayak and Chatterjee, 2003). In the presence of L-GAD, L-glutamic acid is transformed into GABA which absorbs at 455 nm. Into dry tubes, 1 mL of homogenate and 1 ml of glutamic acid (0.05 M; pH 6.7) were introduced, and then 0.1 mL of pyridoxal-5-phosphate (50 mM). The mixture was incubated at 37°C for 30 min, then at 60°C for 10 min. The absorbance of GABA formed in the mixture was read at 455 nm, at 30 s and 90 s against the blank. The activity of L-GAD was estimated in pg of GABA produced/min/mg of fresh tissue.

#### 2.7.4 Determination of reduced glutathione level

The estimation of GSH was carried out according to previous protocols (Rahman et al., 2006). 2,2′-Dithio-5,5′-dinitrobenzoic acid (DTNB) reacts with the–SH groups of glutathione to form a yellow-colored complex that absorbs at 412 nm. To carry out this assay, 1.5 mL of Ellman’s reagent was introduced into test tubes, previously containing 100 μL of homogenate (test tubes), and into a blank tube containing 100 μL of Tris buffer (50 mM HCl; 150 mM KCl; pH 7.4). Tubes were then shaken and incubated for 1 hour at room temperature. The absorbance of the formed yellow-colored complex was read at 412 nm against the blank. The amount of GSH was expressed in µmol/g tissue.

#### 2.7.5 Determination of malondialdehyde level

The MDA level was determined according to the method described by Wilbur et al. (1949). Thiobarbituric acid reacts with MDA and produces a pink-colored complex that can be detected at 530 nm. To perform this assay, 250 µL of homogenate was introduced into the test tubes, while 250 µL of Tris buffer (50 mM HCl; 150 mM KCl; pH 7.4) was introduced into the blank tube. To each tube was then added 125 µL of 20% trichloroacetic acid, then 250 µL of 0.67% thiobarbituric acid. The tubes were plugged with glass beads and then incubated for 10 min at 90°C in a water bath. Thereafter, they were left in the open air for cooling before being centrifuged at 3,000 rpm for 15 min at room temperature (25°C–27°C). The supernatant was removed and the absorbance of the formed complex was read at 530 nm against the blank. The level of MDA was expressed in mmol ⁄g tissue.

#### 2.7.6 Determination of superoxide dismutase activity

The evaluation of SOD activity was performed according to the method of Misra and Fridovish (1972). The presence of SOD in the sample inhibits the oxidation of adrenaline to adrenochrome which absorbs at 480 nm. The control tube contained 1,666 µL of carbonate buffer (50 mM; pH 10.2) only, while in assay tubes contained 134 µL of homogenate. The reaction was triggered by adding 200 µL of 0.3 mM adrenaline solution. After rapid inversion for mixing, the optical density was read at 480 nm at 20 s and 80 s. The specific activity of SOD was expressed in units of SOD/mg of tissue.

#### 2.7.7 Determination of pro-inflammatory cytokine levels

The level of pro-inflammatory cytokines such as tumor necrosis factor-alpha (TNF-α), interleukin one beta (IL1-β), and interleukin six (IL-6) was quantified using the enzyme-linked immunosorbent assay (ELISA). All experiments were performed according to manufacturer instructions (Quantikine ELISA kits, R and D systems, Inc, Minneapolis, United States). The absorbance of each cytokine was measured at 450 nm with a microplate reader. The level of each cytokine, expressed in pg/mL, was determined from the corresponding calibration curve.

### 2.8 Statistical analysis

Statistical analysis of the values obtained and the construction of graphs were performed using Graph Pad Prism version 8.03 and Microsoft Office Excel 2019. The results are expressed as the mean ± standard error of the mean (SEM) or as percentages.

The normality of the data was assessed using Kolmogorov-Smirnov and Shapiro-Wilk tests, while the homogeneity of variance between experimental groups was determined by the test of Brown-Forsythe. When the assumption of the homogeneity of variance was confirmed, the one-way ANOVA test was performed. Thus, if the difference between experimental groups was established, the Tukey *post-hoc* test was therefore performed. Moreover, when the data did not assume Gaussian distribution, the test of Kruskal Wallis followed by the *post-hoc* test of Dunn test was done. The differences were considered significant from *p* < 0.05.

## 3 Histological analysis of the hippocampus

Histological analysis followed the protocol of Survana et al. (2019). The steps included fixation, trimming, dehydration, inclusion, cutting, staining, mounting, and observation. Following the sacrification, animals were killed, and the brains were removed and kept in 4% formalin for 1 week. The brain tissues were cut, placed in labeled cassettes, and immersed for 1 h in a bath of ethanol 70%. The tissues were then cleaned of all traces of water before inclusion in paraffin. For this purpose, the tissues were left in ethanol baths of increasing concentration, i.e., ethanol at 70% (1 h), 95% (1 h), 95% (1 h 30 min), 100% (1 h), 100% (1 h 30), 100% (2 h), respectively. Brain histology was performed by embedding 5-μm sections of tissue in the paraffin, and three sections per mouse of the hippocampus (composed of CA1, CA2, and CA3 layers) were processed. The cutting of the paraffin blocks was performed using a microtome. Then, the blocks of paraffin were recovered with glass slides, and dried in an oven at 45°C for 24 h before staining with hematoxylin-eosin. The slides were stained with hematoxylin and eosin, mounted, and observed at 200× magnifications using an optical microscope (Scientico STM-50, Scientico Medico Engineering Instruments., Haryana, India). This microscope was equipped with a digital camera (Celestron brand 44421) connected to a computer. The obtained images were analyzed with Image J software (version 1.5) to count the number of surviving neurons per µm^2^. Indeed, three slides per sample were analyzed, and in each slide, the number of neurons was counted in four quadrants. Thus, four fields per quadrant were taken for measurement. These surviving neurons were differentiated from other cells such as glial cells in terms of structure and staining. Surviving neurons (pyramidal neurons) are the biggest cells of the brain tissues and possess dendrites and axons. Oligodendrocytes are littler and darker than neurons, while astrocytes have the same size as neurons but lack axons and dendrites. Furthermore, degenerated/necrotic neurons were characterized by a dark cytoplasm, pyknotic nucleus, perineuronal edema, shrunken cytoplasm, and karyolysis.

## 4 Quantitative phytochemical assays

Total phenolic, condensed tannins, flavonoid contents, total alkaloids, and total saponins were respectively quantified using methods described by Hatami et al. (2014), Dehpour et al. (2009), Mang’ Dobara et al. (2020), Ba et al. (2010), and Gracelin et al. (2013).

## 5 Results

### 5.1 The aqueous extract of *P. biglobosa* reduced the mean number of seizures during the PTZ-kindling process

**FIGURE 2:**
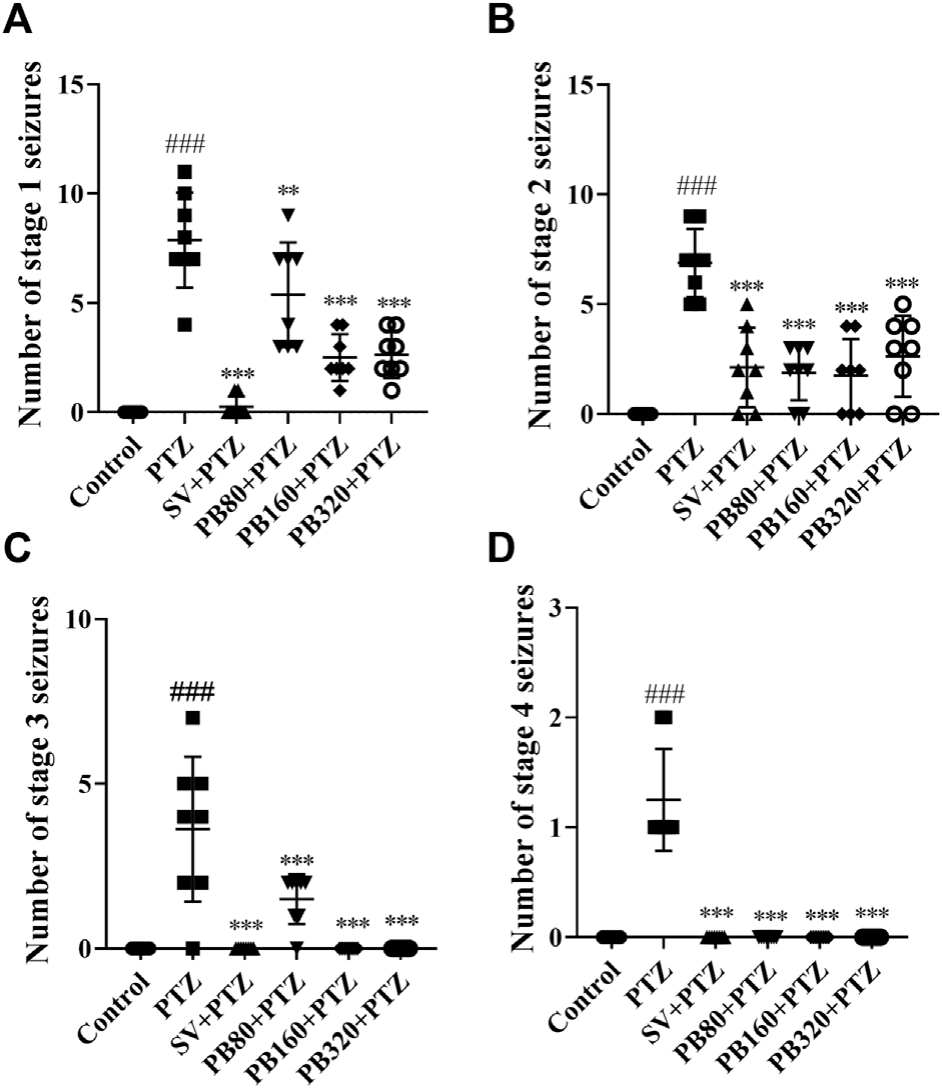
The aqueous extract of *P. biglobosa* decreased the mean number of seizures of stage 1 **(A)** and stage 2 **(B)**. Moreover, it protected the animals against seizures of stage 3 **(C)** and stage 4 **(D)**. Results are expressed as mean percentage ± SEM, *N* = 8. Data were analyzed by one-way ANOVA, followed by Tukey (HSD). ###*p* < 0.001; ##*p* < 0.01 compared to the control group and ***p < 0.001; **p < 0.01; *p < 0.05 compared to the PTZ group. Control, normal control group only treated with distilled water; PTZ = negative control group treated with distilled water and PTZ; PB80, PB160, PB320 + PTZ, test groups treated with the aqueous extract of *P. biglobosa* (80, 160, and 320, respectively) and PTZ; SV + PTZ, positive control group treated with sodium valproate and PTZ. PTZ, pentylenetetrazole. SV, sodium valproate; PB, *Parkia biglobosa*.

Kindling with PTZ increased the number of stages 1, 2, 3, and 4 of seizures in the PTZ group as compared to the control group that did not receive PTZ (Figures 2A–D). The extract, at all doses, decreased (*p* < 0.001) the number of stages 1 and 2 of seizures (Figures 2A,B). However, the extract at all doses completely protected the animals against stages 3 and 4 of seizures (Figures 2C,D). Similarly, sodium valproate led to a decrease in the number of stages 1 and 2 of seizures (Figures 2A,B), and complete protection against stages 3 and 4 seizures (Figures 2C,D).

### 5.2 The aqueous extract *P. biglobosa* decreased the mean duration of seizures during the PTZ-kindling process

**FIGURE 3:**
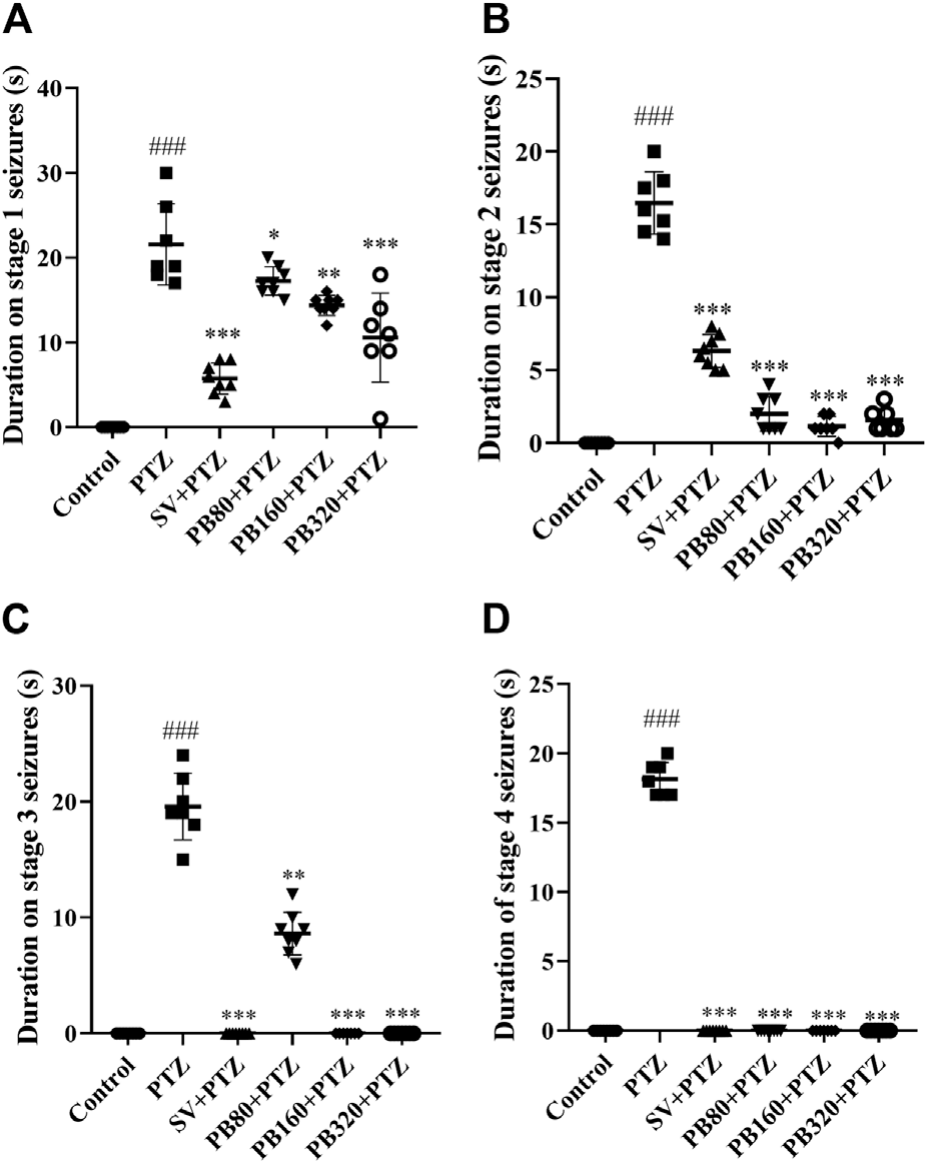
The aqueous extract of *P. biglobosa* decreased the mean duration of seizures of stage 1 **(A)** and stage 2 **(B)**. Seizures of stage 3 **(C)** and stage 4 **(D)** were not observed. Results are expressed as mean percentage ± SEM, *N* = 8. Data were analyzed by one-way ANOVA, followed by Tukey (HSD). ###*p* < 0.001; ##*p* < 0.01 compared to the control group and ***p < 0.001; **p < 0.01; *p < 0.05 compared to the PTZ group. Control, normal control group only treated with distilled water; PTZ = negative control group treated with distilled water and PTZ; PB80, PB160, PB320 + PTZ = test groups treated with the aqueous extract of *P. biglobosa* (80, 160, and 320, respectively) and PTZ; SV + PTZ, positive control group treated with sodium valproate and PTZ. PTZ, pentylenetetrazole. SV, sodium valproate; PB, *Parkia biglobosa*.

The effect of the aqueous extract of *P. biglobosa* extract on seizure duration is shown in Figure 3. The duration of stages 1, 2, 3, and 4 of seizures in the PTZ group increased (*p* < 0.001) as compared to the control group. The extract at all doses, as well as the sodium valproate, decreased (*p* < 0.001) the duration of the seizures of stages 1 and 2. However, these doses of the extract as well as sodium valproate prevented the occurrence of stages 3 and 4 of seizures (Figures 3C,D).

### 5.3 The aqueous extract of *P. biglobosa* improved the recognition of new object during the object recognition task

**FIGURE 4:**
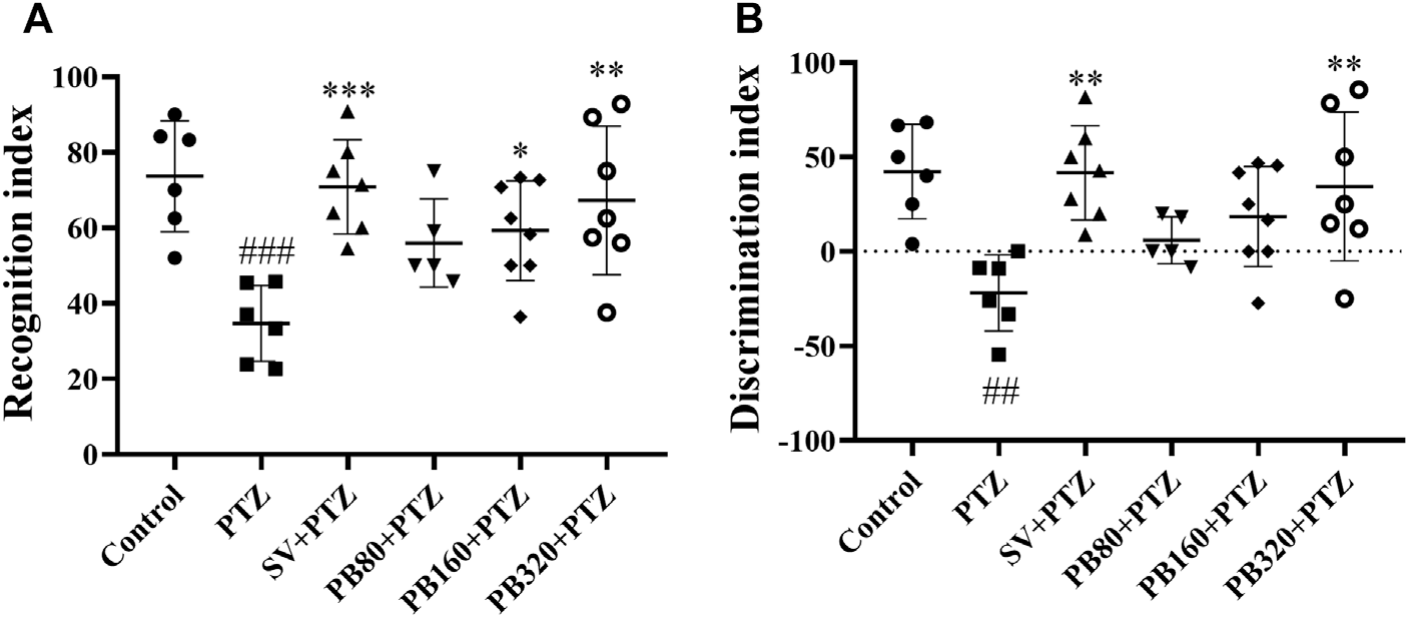
The aqueous extract of *P. biglobosa* increased both the recognition index **(A)** and discrimination index **(B)** during the object recognition test. Results are expressed as mean percentage ± SEM, *N* = 8. Data were analyzed by one-way ANOVA, followed by Tukey (HSD). ###*p* < 0.001; ##*p* < 0.01 compared to the control group and ***p < 0.001; **p < 0.01; *p < 0.05 compared to the PTZ group. Control, normal control group only treated with distilled water; PTZ, negative control group treated with distilled water and PTZ; PB80, PB160, PB320 + PTZ, test groups treated with the aqueous extract of *P. biglobosa* (80, 160, and 320, respectively) and PTZ; SV + PTZ, positive control group treated with sodium valproate and PTZ. PTZ, pentylenetetrazole. SV, sodium valproate; PB, *Parkia biglobosa*.

The effect of the aqueous extract of *P. biglobosa* extract on the recognition or discrimination index is shown in Figure 4. In the PTZ group, the recognition index (Figure 4A) decreased (34.70 ± 5.20; *p* < 0.001) as compared to the control group (73.70 ± 3.80). This activity was decreased in all the test groups, with the greatest effect observed at the dose of 320 mg/kg (67.20 ± 7.50; *p* < 0.01). Likewise, sodium valproate decreased (*p* < 0.001) this index to 70.80 ± 4.40. The discrimination index (Figure 4B) decreased in the PTZ group (−21.90 ± 7.60; *p* < 0.01) relative to the control group (42.30 ± 7.10). The aqueous extract of *P. biglobosa* at the dose of 320 mg/kg and sodium valproate increased this index to 34.50 ± 7.30 (*p* < 0.01) and 41.70 ± 8.80 (*p* < 0.01), respectively.

### 5.4 The aqueous extract of *P. biglobosa* ameliorated the working memory-like behavior in the T-maze task

**FIGURE 5:**
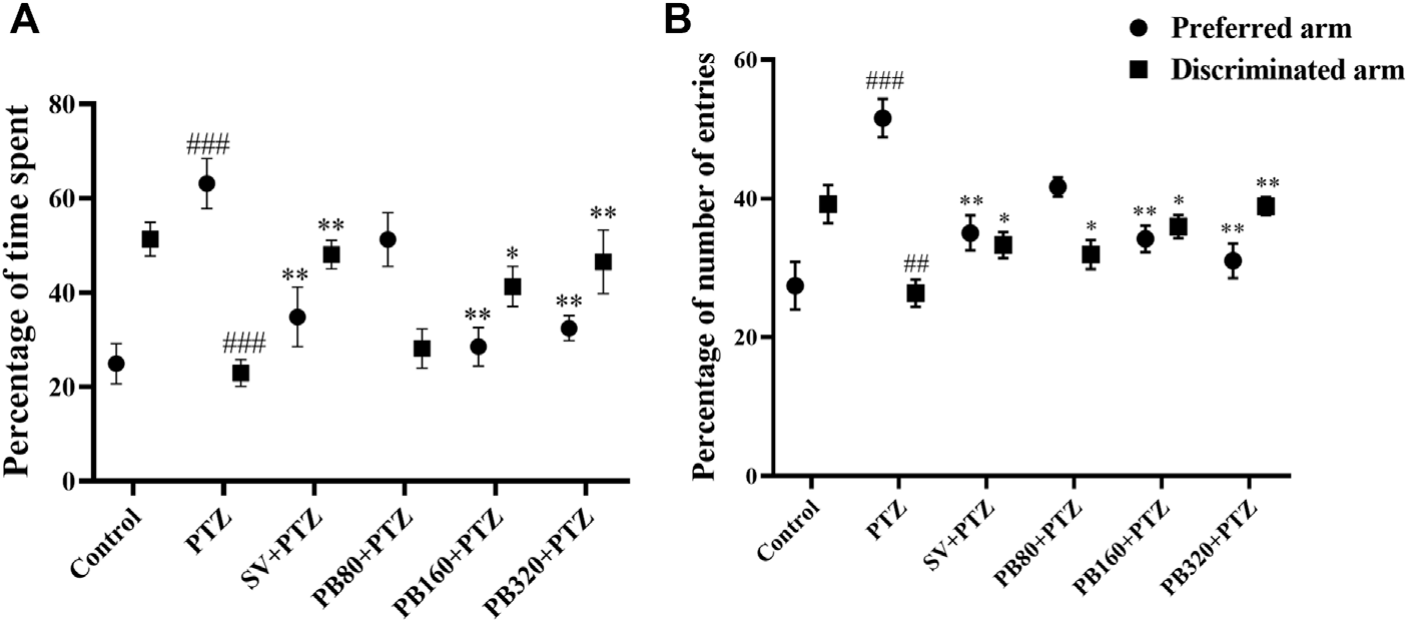
The aqueous extract of *P. biglobosa* increased both the percentage of time spent **(A)** and the percentage number of entries in the discriminated arm **(B)** in the T-maze test. Results are expressed as mean percentage ± SEM, *N* = 8. Data were analyzed by one-way ANOVA, followed by Tukey (HSD). ###*p* < 0.001; ##*p* < 0.01 compared to the control group and **p < 0.01; *p < 0.05 compared to the PTZ group. Control, normal control group only treated with distilled water; PTZ, negative control group treated with distilled water and PTZ; PB80, PB160, PB320 + PTZ, test groups treated with the aqueous extract of *P. biglobosa* (80, 160, and 320, respectively) and PTZ; SV + PTZ, positive control group treated with sodium valproate and PTZ. PTZ, pentylenetetrazole. SV, sodium valproate; PB, *Parkia biglobosa*.

Figure 5A shows the effects of *P. biglobosa* on the percentage of time spent in the preferred arm and the discriminated arm of the T-maze. In the PTZ group, the percentage of time spent decreased (*p* < 0.001) in the preferred arm (63.10 ± 5.30%), and increased (*p* < 0.001) in the discriminated arm (22.90 ± 2.90%) as compared to the control group (24.90 ± 3.43% in the preferred arm and 53.30 ± 3.60% in the discriminated arm) (Figure 5A). The extract at the dose of 320 mg/kg markedly increased (*p* < 0.01) this percentage in the preferred arm (32.40 ± 2.40%), and decreased (*p* < 0.01) this percentage in the discriminated arm (46.00 ± 6.80%). Similar effects were observed in animals treated with sodium valproate both in the preferred (33.80 ± 6.30%; *p* < 0.01) and discriminated (48.10 ± 3.10%; *p* < 0.05) arms (Figure 5A).

Figure 5B shows the effects of *P. biglobosa* on the percentage number of entries in the preferred arm and the discriminated arm of the T-maze. In the PTZ group, the percentage of the number of entries increased (*p* < 0.001) in the preferred arm (51.60 ± 42.80%) and decreased (*p* < 0.01) in the discriminated arm (26.40 ± 2.00%) as compared to the control group (27.40 ± 3.40% in the preferred arm and 39.20 ± 3.70% in the discriminated arm). The extract at the dose of 320 mg/kg increased (*p* < 0.01) this time in the preferred arm (31.00 ± 2.50%) while it decreased (*p* < 0.01) in the discriminated arm (38.90 ± 1.30%). Similar effects were observed with sodium valproate in the preferred (35.00 ± 2.50%; *p* < 0.01) and discriminated (33.30 ± 1.90%; *p* < 0.05) arms (Figure 5B).

### 5.5 The aqueous extract of *P. biglobosa* attenuated anxiety-like behavior in the open field test

Table 1 shows the effect of the aqueous extract of *P. biglobosa* on the number of recoveries and time spent in the center as well as the number of rearings in the open field test.

**TABLE 1:** The aqueous extract of *P. biglobosa* reduced anxiety-like behavior in the open field test.

Treatments	Number of recoveries	Time spent at the center (s)	Number of rearings
**Control**	10.90 ± 1.66	16.30 ± 2.28	31.40 ± 2.43
**PTZ**	3.71 ± 0.92^##^	7.17 ± 0.48^##^	60.60 ± 9.39###
**SV + PTZ**	10.40 ± 0.95**	15.60 ± 1.17**	31.70 ± 0.81***
**PB 80 + PTZ**	6.50 ± 1.09	11.00 ± 1.66	38.60 ± 6.86**
**PB 160 + PTZ**	8.43 ± 1.32	13.30 ± 1.06	36.00 ± 3.44**
**PB 320 + PTZ**	9.50 ± 0.96*	14.70 ± 1.48*	31.40 ± 4.48***

Results were expressed as mean ± SEM, *N* = 5. Data were analyzed by one-way ANOVA, followed by Tukey (HSD). ###*p* < 0.001, ##*p* < 0.01 compared to the control group and ∗∗∗*p* < 0.001, ∗∗*p* < 0.01, ∗*p* < 0.05 compared to the PTZ, group. Control = normal control group only treated with distilled water; PTZ, negative control group treated with distilled water and PTZ; PB80, PB160, PB320 + PTZ, test groups treated with the aqueous extract of *P. biglobosa* (80, 160, and 320, respectively) and PTZ; SV + PTZ, positive control group treated with sodium valproate and PTZ. PTZ, pentylenetetrazole. SV, sodium valproate; PB, *Parkia biglobosa*.

The number of recoveries decreased (*p* < 0.01) in the PTZ group (3.70 ± 0.90) as compared to the control group (10.90 ± 1.66). The number of recoveries significantly increased both in the aqueous extract at the dose of 320 mg/kg (9.50 ± 1.00; *p* < 0.05) and sodium valproate group (10.40 ± 1.00; *p* < 0.01). The time spent at the center was lower (*p* < 0.01) in the PTZ group (7.20 ± 0.50 s) as compared to the control group (16.30 ± 2.30 s) (Table 1). The aqueous extract increased this time with the greatest effect at the dose of 320 mg/kg (14.70 ± 1.50 s; *p* < 0.05). Similarly, sodium valproate increased (15.80 ± 1.20 s; *p* < 0.01) this time (Table 1).

The number of rearings was higher (*p* < 0.001) in the PTZ group (60.60 ± 9.40) as compared to the control group (31.40 ± 2.40) (Table 1). The aqueous extract decreased this number with the greatest effect at the dose of 320 mg/kg (31.40 ± 4.50; *p* < 0.001). Sodium valproate also decreased this number (31.70 ± 0.80; *p* < 0.01) (Table 1).

### 5.6 The aqueous extract of *P. biglobosa* increased the level of GABA in the hippocampus

**FIGURE 6:**
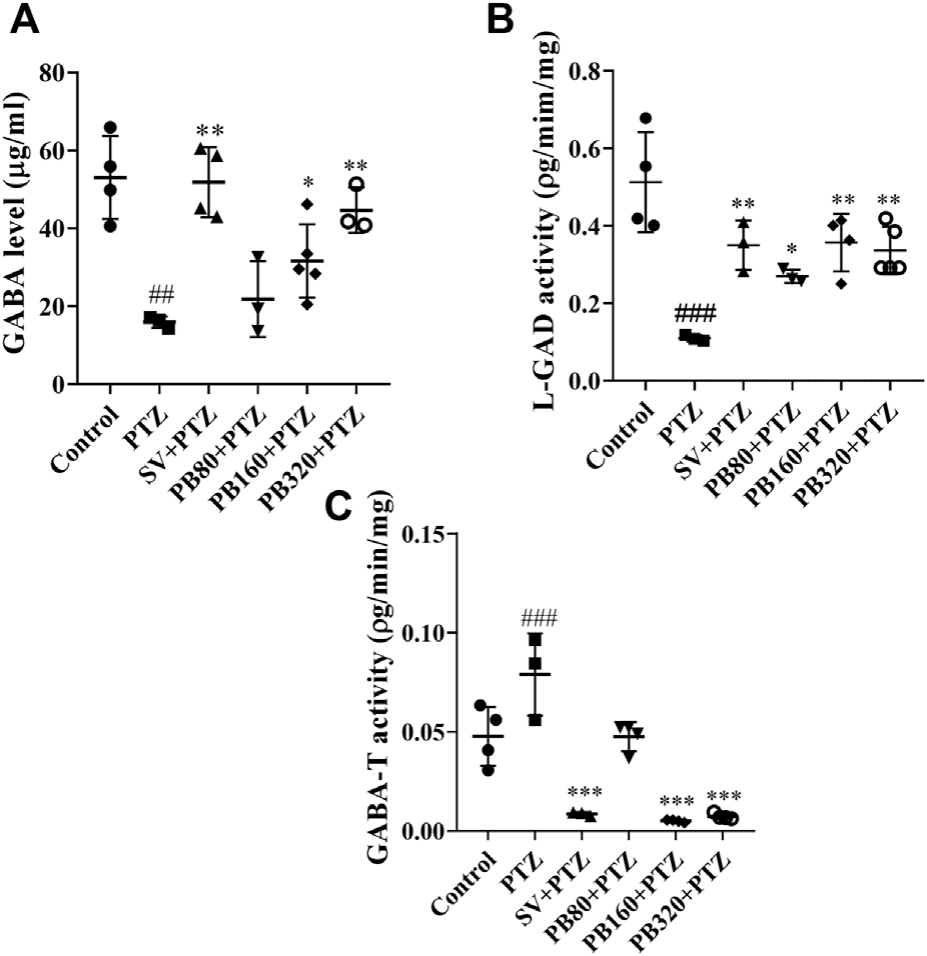
The aqueous extract of *P. biglobosa* increased the levels of GABA **(A)** and L-GAD **(B)** and decreased GABA-T activity **(C)**. Results are expressed as mean ± SEM, *N* = 5. Data were analyzed by one-way ANOVA, followed by Tukey (HSD). ###*p* < 0.001; ##*p* < 0.01 compared to the control group and ***p < 0.001; **p < 0.01; *p < 0.05 compared to the PTZ group. Control = normal control group only treated with distilled water; PTZ, negative control group treated with distilled water and PTZ; PB80, PB160, PB320 + PTZ, test groups treated with the aqueous extract of *P. biglobosa* (80, 160, and 320, respectively) and PTZ; SV + PTZ, positive control group treated with sodium valproate and PTZ. PTZ, pentylenetetrazole. SV, sodium valproate; PB, *Parkia biglobosa*.

The effects of the *P. biglobosa* extract on the GABA metabolism parameters (GABA, L-GAD, and GABA-T) in the hippocampus are shown in Figure 6. The GABA level was reduced (*p* < 0.01) in the PTZ group (15.90 ± 0.70 μg/ml) as compared to the control group (53.00 ± 4.40 μg/ml) (Figure 6A). The extract at all doses significantly increased this level. However, the 320 mg/kg dose markedly increased this level (44.70 ± 2.90 μg/ml; *p* < 0.01). Likewise, sodium valproate increased this level (51.20 ± 5.40 μg/ml; *p* < 0.01) (Figure 6A). The L-GAD activity decreased (*p* < 0.001) in the PTZ group (0.11 ± 0.04 pg/min/mg) as compared to the control group (0.51 ± 0.06 pg/min/mg) (Figure 6B). The extract of *P. biglobosa* at the dose of 160 mg/kg (dose which showed the most effective effect) as well as sodium valproate, increased the activity to 0.36 ± 0.02 pg/min/mg (*p* < 0.01) and 0.35 ± 0.03 pg/min/mg (*p* < 0.01), respectively (Figure 6B). The GABA-T activity increased (*p* < 0.001) in the PTZ group (0.08 ± 0.01 pg/min/mg) as compared to the control group (0.05 ± 0.01 pg/min/mg) (Figure 6C). This activity was decreased in all the test groups (*p* < 0.001), with the greatest effect observed at the dose of 160 mg/kg (0.01 ± 0.00 pg/min/mg). Likewise, sodium valproate decreased this activity to 0.02 ± 0.00 pg/min/mg (*p* < 0.001) (Figure 6C).

### 5.7 The aqueous extract of *P. biglobosa* reduced oxidative stress markers in the hippocampus

The effect of the aqueous extract *P. biglobosa* on the levels of MDA, GSH, and SOD in the hippocampus is shown in Table 2. MDA level increased (*p* < 0.001) in the PTZ group (17.90 ± 0.56 pmol/g) as compared to the control group (6.10 ± 0.50 pmol/g). The aqueous extract of *P. biglobasa,* at the dose of 320 mg/kg (dose which showed the greatest effect), as well as sodium valproate, decreased (*p* < 0.001) this level to 10.70 ± 0.40 pmol/g and 12.40 ± 0.50 pmol/g, respectively (Table 2). GSH level decreased (*p* < 0.001) in the PTZ group (201.60 ± 1.00 μmol/g) as compared to the control group (818.20 ± 1.20 μmol/g). The aqueous extract of *P. biglobosa* at the dose of 320 mg/kg (dose which showed a marked effect), as well as sodium valproate, increased (*p* < 0.001) this level to 730.20 ± 0.80 μmol/g and 673.50 ± 0.90 μmol/g, respectively (Table 2). SOD activity decreased (*p* < 0.001) in the PTZ group (306.20 ± 5.10 unit/min/g) as compared to the control group (476.40 ± 7.40 unit/min/g). The aqueous extract of *P. biglobosa* at the dose of 320 mg/kg (dose with the greatest effect), as well as sodium valproate, increased (*p* < 0.001) this level to 425.30 ± 4.20 unit/min/g and 491.30 ± 3.20 unit/min/g, respectively (Table 2).

**TABLE 2:** The aqueous extract of *P. biglobosa* reduced oxidative stress markers parameters in the hippocampus.

Treatments	MDA (pmol/g)	GSH (µmol/g)	SOD (Unit/min/g)
Control	6.10 ± 0.54	818.24 ± 1.22	476.42 ± 7.37
PTZ	17.90 ± 0.56###	201.58 ± 0.95###	306.16 ± 5.14###
SV + PTZ	12.40 ± 0.51***	673.51 ± 0.92**	491.33 ± 3.18***
PB 80 + PTZ	13.80 ± 0.74**	301.92 ± 0.86	417.45 ± 6.41*
PB 160 + PTZ	13.60 ± 0.69**	479.64 ± 0.63*	425.28 ± 4.22*
PB 320 + PTZ	10.70 ± 0.35***	730.15 ± 0.80***	458.15 ± 3.48**

Results are expressed as mean ± SEM, *N* = 5. Data were analyzed by one-way ANOVA, followed by Tukey (HSD). ###*p* < 0.001 compared to the control group and ∗∗∗*p* < 0.001, ∗∗*p* < 0.01, ∗*p* < 0.05 compared to the PTZ, group. Control, normal control group only treated with distilled water; PTZ, negative control group treated with distilled water and PTZ; PB80, PB160, PB320 + PTZ, test groups treated with the aqueous extract of *P. biglobosa* (80, 160, and 320, respectively) and PTZ; SV + PTZ, positive control group treated with sodium valproate and PTZ. PTZ, pentylenetetrazole. SV, sodium valproate; PB, *Parkia biglobosa*.

### 5.8 The aqueous extract of *P. biglobosa* decreased the levels of pro-inflammatory cytokine markers in the hippocampus

**FIGURE 7:**
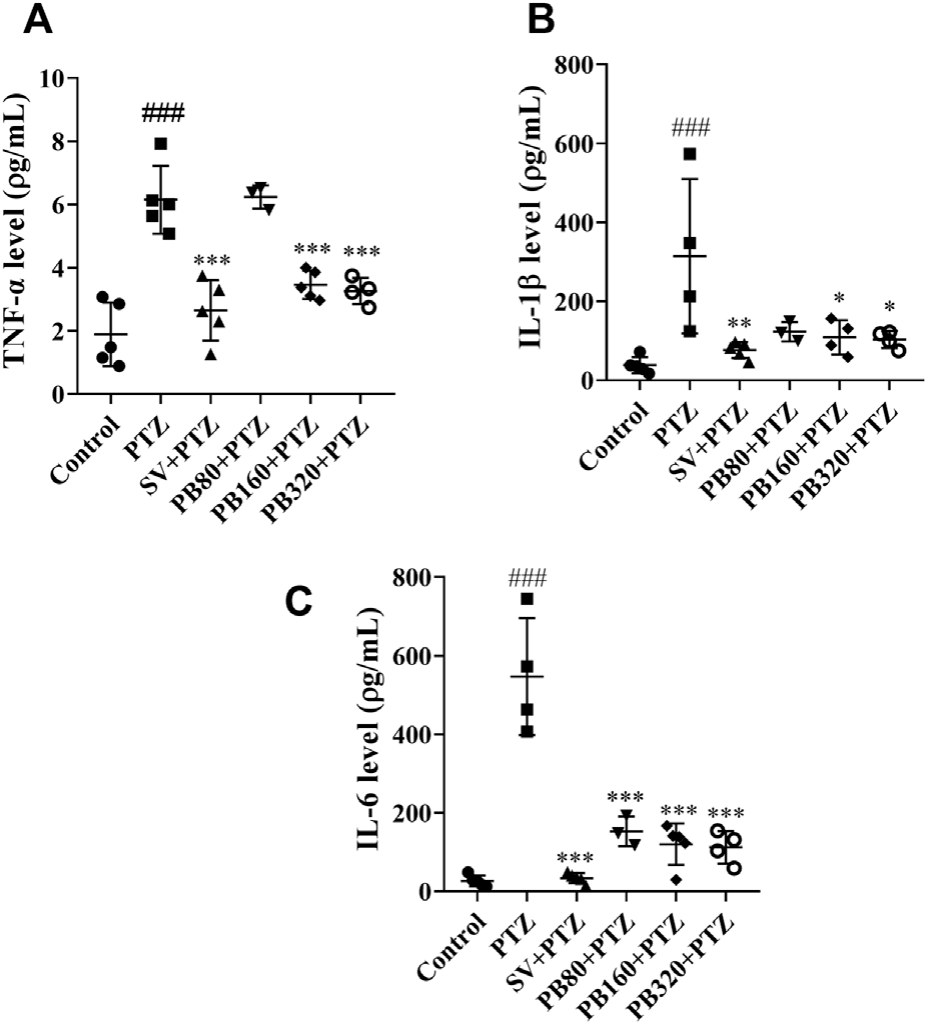
The aqueous extract of *P. biglobosa* decreased the levels of TNF-α **(A)**, IL-1β **(B)**, and IL-6 **(C)** in the hippocampus. Results are expressed as mean ± SEM, *N* = 5. Data were analyzed by one-way ANOVA, followed by Tukey (HSD). ###*p* < 0.001 compared to the control group and ***p < 0.001; **p < 0.01; *p < 0.05 compared to the PTZ group. Control = normal control group only treated with distilled water; PTZ = negative control group treated with distilled water and PTZ; PB80, PB160, PB320 + PTZ = test groups treated with the aqueous extract of *P. biglobosa* (80, 160, and 320, respectively) and PTZ; SV + PTZ = positive control group treated with sodium valproate and PTZ. PTZ, pentylenetetrazole. SV, sodium valproate; PB, *Parkia biglobosa*.

Figure 7 shows the effects of the aqueous extract of *P. biglobosa* on TNF-α, IL-1β, and IL-6 levels. The TNF-α level increased to 6.20 ± 0.50 pg/mL (*p* < 0.001) in the PTZ group as compared to the control group (1.90 ± 0.40 pg/ml) (Figure 7A). The aqueous extract at the doses of 160 and 320 increased (*p* < 0.001) the level of this cytokine. Likewise, sodium valproate decreased (*p* < 0.001) this level to 2.60 ± 0.40 pg/mL (Figure 7A). The level of IL-1β increased (*p* < 0.001) in the PTZ group (314.30 ± 97.70 pg/ml) as compared to the control group (38.20 ± 9.20 pg/ml). The aqueous extract of *P. biglobosa* at doses of 160 and 320 mg/kg decreased the level of cytokine. The maximal effect was observed at the dose of 320 mg/kg (103.40 ± 10.80 pg/ml; *p* < 0.05). Sodium valproate decreased (*p* < 0.01) this level to 76.60 ± 9.10 pg/ml (Figure 7B). The level of IL-6 increased (*p* < 0.001) in the PTZ group (546.70 ± 74.40 pg/ml) as compared to the control group (26.90 ± 6.30 pg/ml) (Figure 7C). The aqueous extract of *P. biglobosa* at all doses markedly decreased the level of this cytokine. The maximal effect was observed at the dose of 320 mg/kg (112.20 ± 20.60 pg/ml; *p* < 0.001). Sodium valproate equally decreased (*p* < 0.001) this level to 34.10 ± 5.70 pg/ml (Figure 7C).

### 5.9 The aqueous extract of *P. biglobosa* attenuated degeneration/necrosis of pyramidal neurons in CA1, CA2, and CA3 regions of the hippocampus

**FIGURE 8:**
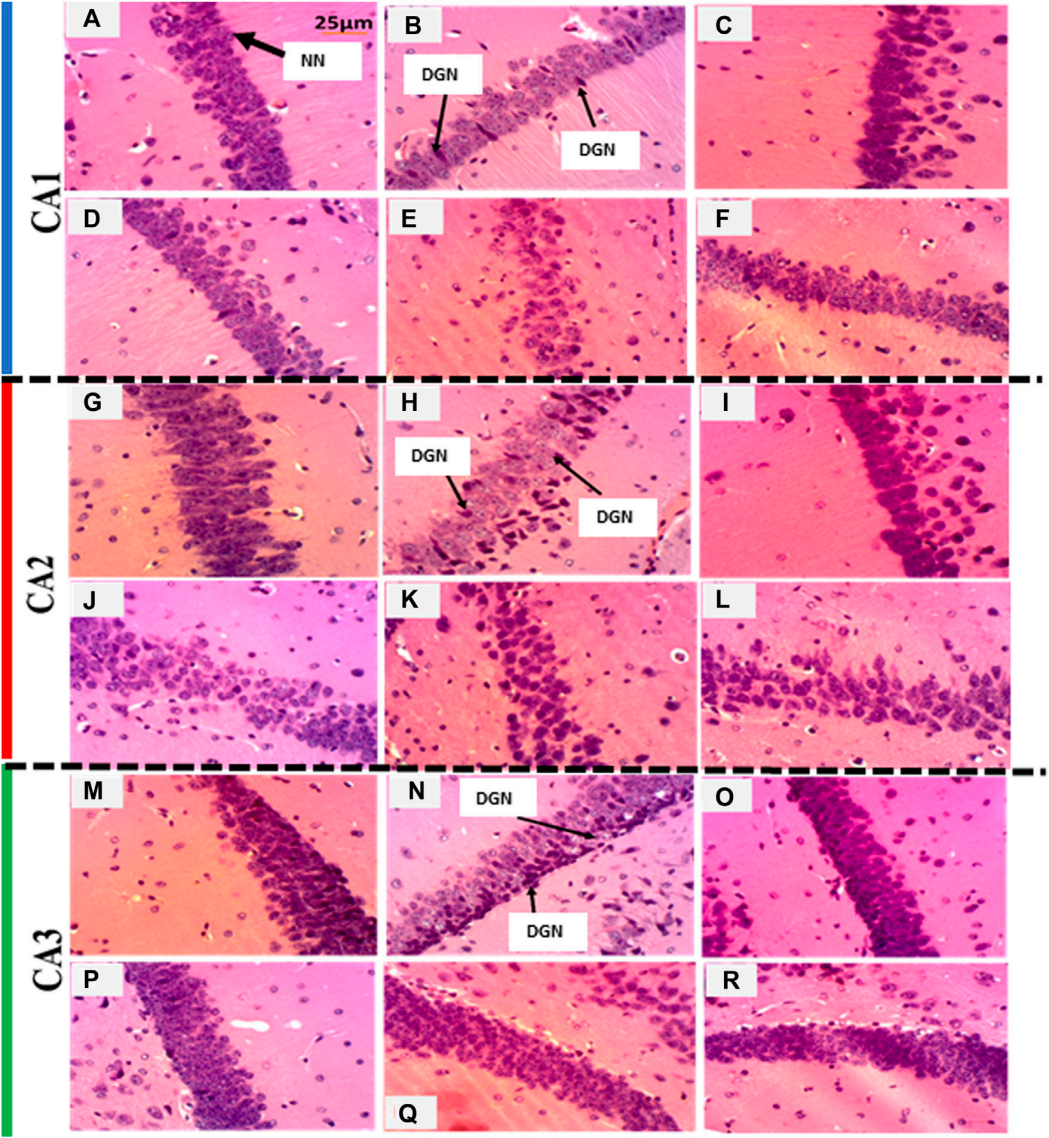
The aqueous extract of *P. biglobosa* attenuated degenerated/necrotic neurons in CA1, CA2, and CA3 regions of the hippocampus after hematoxylin-eosin staining (200 ×). Scale: 25 µm. DGN: degenerated neurons; NN: normal neurons; Panels **(A,G,M)**: normal control group; Panels B, H, and N: negative control group; Panels **(C,I,O)**: sodium valproate group; Panels **(D,J,P)**: group treated with the extract of *P. biglobosa* at the dose of 80 mg/kg; Panels **(E,K,Q)**: group treated with the extract of *P. biglobosa* at the dose of 160 mg/kg; Panels **(F,L,R)**: group treated with the extract of *P. biglobosa* at the dose of 320 mg/kg.

Figure 8 shows the effects of the aqueous extract of *P. biglobosa* on the microarchitecture of the *Cornus Ammonis* (CA) regions of the hippocampus. Compared to the control group (Figures 8A,G,M), degenerated/necrotic neurons were observed in the PTZ group (Figures 8B,H,N). These alterations were confirmed by a reduction (*p* < 0.01) in the number of neurons in the CA1, CA2, and CA3 (Table 3). At the dose of 80 mg/kg of the extract, there were few degenerated/necrotic neurons (Figure 8D), and an increased number (*p* < 0.05) of surviving neurons in the CA1 region (Table 3). However, in CA2 and CA3 regions, the extract at all doses (Figures 8 J,K,L,P,Q,R), as well as sodium valproate (Figures 8I,O) reduced the presence of degenerated/necrotic neurons (Figures 8J,K,L). These treatments also increased (*p* < 0.05–0.01) the number of surviving neurons (Table 3).

**TABLE 3:** The aqueous extract of *P. biglobosa* reduced degenerated/necrotic neurons in the hippocampus.

Treatments	CA1 (Nb/µm^2^)	CA2 (Nb/µm^2^)	CA3 (Nb/µm^2^)
Control	143.56 ± 0.43	156.11 ± 0.86	132.40 ± 0.25
PTZ	84.11 ± 0.11#	56.03 ± 0.77##	60.12 ± 0.59##
SV + PTZ	67.33 ± 0.30	100.64 ± 0.74*	123.55 ± 0.68**
PB 80 + PTZ	122.15 ± 0.66*	123.11 ± 0.55*	108.37 ± 0.75*
PB 160 + PTZ	65.50 ± 0.52	42.33 ± 0.51	129.48 ± 0.68**
PB 320 + PTZ	101.55 ± 0.44	116.29 ± 0.70*	119.84 ± 0.90*

Results were expressed as mean ± SEM, *N* = 3. Data were analyzed by one-way ANOVA, followed by Tukey (HSD##*p* < 0.01 compared to the control group and ∗∗*p* < 0.01, ∗*p* < 0.05 compared to the PTZ, group. Control, normal control group only treated with distilled water; PTZ, negative control group treated with distilled water and PTZ; PB80, PB160, PB320 + PTZ, test groups treated with the aqueous extract of *P. biglobosa* (80, 160, and 320, respectively) and PTZ; SV + PTZ, positive control group treated with sodium valproate and PTZ. PTZ, pentylenetetrazole; SV, sodium valproate; PB, *Parkia biglobosa*; CA1-3 = *Cornu ammonis* layers 1, 2, and 3. Nb, number of surviving neurons.

### 5.10 Quantitative phytochemical analysis

Total phenolic compounds (88.12 ± 0.15 mg GAE/g), condensed tannins (27.00 ± 0.13 mg CatE/g), and flavonoids contents (43.66 ± 0.35 mg RE/g) were abundant as compared to standards (Table 4). However, total alkaloids (13.06 ± 0.11%) and saponins (4.59 ± 0.31%) were less abundant (Table 4).

**TABLE 4:** Quantitative analysis of the aqueous extract of *P. biglobosa*.

Chemical compounds unit	Mean
**Alkaloids %**	13.06 ± 0.11%
**Flavonoids mg RE/g**	43.66 ± 0.35
**Tannins mg CatE/g**	27.00 ± 0.13
**Polyphenols mg GAE/g**	88.12 ± 0.15
**Saponins %**	4.59 ± 0.31

RE, rutin equivalent; CatE, catechin equivalent; GAE, gallic acid equivalent; %, percentage.

## 6 Discussion

Epilepsy is a chronic brain disorder in need of more effective therapies. Indeed, the failure of conventional antiepileptogenic drugs to effectively inhibit the epileptogenic process has paved the way for the discovery of new therapies (Okaiyeto and Oguntibeju, 2021). The kindling model closely mimics neuropathological conditions associated with generalized seizures (Dhir, 2012; Singh et al., 2021). Recent studies showed that PTZ can induce epileptic seizures in rodents by selectively blocking the chloride channel of the GABA_A_ receptor (Dhir, 2012; Singh et al., 2021). In the present study, chronic injection of PTZ induced an increase in the number and duration of stages 1, 2, 3, and 4 seizures. These results are similar to those of Cetindag and Ciltas (2022) and Mishra et al. (2021b) who worked on a similar model. The condition in which a normal brain is altered towards the generation of abnormal electrical activity is called epileptogenesis (Dhir, 2012; Singh et al., 2021). Thus, molecules that inhibit or alter kindling processes have the potential to be developed as disease-modifying or antiepileptogenic drugs (Dhir, 2012; Singh et al., 2021). The administration of an aqueous extract of the leaves of *P. biglobosa* significantly reversed the effects and suggests an antiepileptogenic potential. The quantitative analysis of the phytochemical compounds of the extract revealed a high concentration of flavonoids and saponins. The antiepileptogenic-like effect of the extract could be related to the presence of these compounds in the extract (Jiang, 2019; Xia et al., 2021; Yakubu et al., 2021).

During epileptogenesis, memory and emotion are particularly altered because they share the same anatomical structure with TLE. This explains why working memory impairment and anxiety disorders are common complaints in patients with TLE (Kandeda et al., 2021b; Kandeda et al., 2022c; Kandeda et al., 2022d). Thus, a drug that can prevent or inhibit the development of these neuropsychiatric disorders, in people at risk, is of interest (Singh et al., 2021; Pollo et al., 2022). In the present study, PTZ group animals showed working memory impairment in the object recognition and T-maze tests. Vogt et al. (2022) reported similar cognitive and behavioral impairments following a chronic injection of PTZ in rodents. Indeed, PTZ-kindling provides the opportunity to study associated cognitive deficits (Singh et al., 2018; de Souza et al., 2019; Kandeda et al., 2022d). Furthermore, during the open field test, PTZ-kindling led to a decrease in anxiety-like behavior in the PTZ group. Such variation was obtained by Hoeller et al. (2017) and Anesti et al. (2020) and indicates a state of anxiety-like behavior. According to these authors, the inhibition of GABA neurotransmission in the hippocampus and amygdala could be the reason behind these alterations, since these regions are involved in the regulation of anxiety behavior. The extract of *P. biglobosa* reversed all these parameters in the object recognition and T-maze tests as well as in the open-field test. These results are similar to those of Mishra et al. (2021a) and suggest anti-amnesic- and anxiolytic-like properties. These findings confirm the antiepileptogenic potential of the plant extract since working memory impairment, anxiety disorders, and epileptogenesis share similar bidirectional and cause-to-effect links (Shishmanova-Doseva et al., 2021; Suleymanova, 2021).

In the view to unraveling the mechanism of action by which the extract induced these behavioral changes, we began to measure the concentration of GABA. This latter, which is the main inhibitory neurotransmitter in the central nervous system, is a rapid biomarker of molecular alterations during epileptogenesis (Dhir, 2012). Thus, a decrease in GABA neurotransmission is a characteristic of an epileptic condition (Shimada and Yamagata, 2018; Szyndler et al., 2018). Analysis of markers of GABA metabolism in the present study showed that GABA-T activity increased and GABA level decreased in the PTZ group. These data are consistent with those of Erkec et al. (2022) who showed that PTZ-kindling decreased the GABA level in the hippocampus of rats. The aqueous extract of *P. biglobosa* reduced GABA-T activity and increased the GABA level, indicating modulation of GABA neurotransmission. These effects could be related to the presence of flavonoids and saponins. These compounds have been shown to decrease the activity of GABA-T and increase the level of GABA in the brain (Xie et al., 2018; Rebas et al., 2020). Increasing the level of GABA could be the mechanism by which the extract reduces seizures and anxiety disorders as revealed in the present work.

Apart from the involvement of GABA neurotransmission during epileptogenesis, accumulating literature revealed the role of oxidative stress during this process. Thus, a drug with antioxidant potential could contribute to preventing or inhibiting epileptogenesis (Li et al., 2021; Erkec et al., 2022). In the present study, the aqueous extract reduced oxidative stress as compared to the PTZ group. PTZ stimulates the glutamatergic system which is at the origin of the excitotoxicity in the hippocampal neurons. Free radicals that result could cause lipid peroxidation, generating MDA and a drop in GSH. Given that the extract reversed these changes, these findings suggest antioxidant properties and confirm previous studies demonstrating a strong antioxidant potential of *P. biglobosa* extract (Banwo et al., 2020; Musara et al., 2020). Furthermore, flavonoids and polyphenols are known to reduce the formation of reactive oxygen species and free radicals (Sahu et al., 2020; Lazzarotto et al., 2021). The abundance of these secondary metabolites in the extract could explain its antioxidant properties.

Oxidative stress is associated with free radicals synthesis during epileptogenesis (Ghorbanian et al., 2019; Wu et al., 2020), and is generally the consequence or the cause of the inflammatory process in the brain (Ghorbanian et al., 2019; Wu et al., 2020). Thus, treatment with both anti-inflammatory and antioxidant potentials is of interest. Pro-inflammatory cytokines were reported to aggravate seizures in clinical studies (Ghorbanian et al., 2019; Wu et al., 2020). In the present work, an analysis of inflammatory parameters after PTZ-kindling revealed a significant increase in the levels of IL-1β, IL-6, and TNF-α. These results are consistent with those of Temp et al. (2017) and Hoda et al. (2017) who showed a similar variation following chronic PTZ injections in rats. PTZ can activate microglia and induce the release of TNF-α, IL-1β, and IL-6 (Kandeda et al., 2022d). These cytokines can increase glutamate level and stimulate endocytosis of GABA receptors (Temp et al., 2017; Sharma et al., 2021). Administration of the aqueous extract significantly reduced the levels of these pro-inflammatory cytokines, suggesting anti-inflammatory activity. This activity could be one of the mechanisms involved in the antiepileptogenic-like effect of the extract. Quantitative analysis of the extract revealed the presence of a high concentration of metabolites such as alkaloids, flavonoids, tannins, and saponins. These compounds have been shown to act as cerebral anti-inflammatory agents (Qureshi et al., 2011; Malaguarnera, 2019). The anti-inflammatory activity of these compounds could contribute to the antiepileptogenic-like effect of the extract.

Inflammatory processes and oxidative stress are generally associated with brain injury. Hence, a drug combining anti-inflammatory and antioxidant properties can reduce or prevent the degeneration of neurons. In the present work, PTZ-kindling caused degeneration/necrosis in the hippocampus of the PTZ group. These results are in agreement with those of Kandeda et al. (2022b) who revealed the disorganization of the hippocampus of PTZ-kindled rats. The extract of *P. biglobosa* reduced this neuronal degeneration/necrosis, perhaps by the antioxidant and anti-inflammatory activities. In addition, behavioral alterations such as amnesia and anxiety are thought to be the result of histological alterations in the brain. The anti-amnesic- and anxiolytic-like effects of the extract could be due to the neuroprotective effect of the extract (Yahaya et al., 2014; Kandeda et al., 2017; Kandeda et al., 2021a; Kandeda et al., 2022a). This neuroprotective effect could be due to the abundance of flavonoids, polyphenols, and alkaloids in the extract (Abdulwanis et al., 2019;Kandeda et al., 2021c).

## Conclusion

This study was aimed at evaluating the antiepileptogenic-, anti-amnesic, and anxiolytic-like effects of an aqueous extract of *P. biglobosa* in the PTZ-induced kindling model. The aqueous extract of *P. biglobosa* prevented seizures, impairment of working memory, and anxiety-like behavior in mice. These properties were associated with neuromodulatory, antioxidant, and anti-inflammatory activities. The secondary metabolites found in this extract could account for these properties, thus providing rational scientific evidence for its use in folkloric medicine against neurological disorders. Further studies should be conducted to elucidate the molecular mechanisms of action of the aqueous extract of *P. biglobosa*, and the specific molecular components involved using *in vitro* models of epileptogenesis.

## Data Availability

The raw data supporting the conclusion of this article will be made available by the authors, without undue reservation.

## Abbreviations

GABA, γ-aminobutyric acid; GABA-T, γ-aminobutyric acid transaminase; L-GAD: L-glutamate decarboxylase; GSH, reduced glutathione; i.p., intraperitoneally; IL-1β: interleukin 1beta; IL-6, Interleukin 6; MDA, malondialdehyde; SOD, superoxide dismutase; p.o., per os; PB, *Parkia biglobosa* aqueous extract; PB80: *Parkia biglobosa* at the dose of 80 mg/kg; PB160, *Parkia biglobosa* at the dose of 160 mg/kg; PB320, *Parkia biglobosa* at the dose of 320 mg/kg; PB80, *Parkia biglobosa* at the dose of 80 mg/kg; PTZ: pentylenetetrazole; SV, sodium valproate; TLE, temporal lobe epilepsy; TNF-α, tumor necrosis factor-alpha.
